# Effectiveness of a Five-Day Multimodal Intensive Treatment Program for Obsessive-Compulsive Disorder: A Prospective Observational Study

**DOI:** 10.7759/cureus.113867

**Published:** 2026-08-03

**Authors:** Saikumar M. Reddy, Swetha Reddy, Nissi Rufus, Balaji Sainath, Lalitha Jahnavi, Aswin Kumar Mudunuru

**Affiliations:** 1 Department of Psychiatry, Asha Neuromodulation Clinics, Hyderabad, IND; 2 Department of Psychology, Asha Neuromodulation Clinics, Hyderabad, IND; 3 Department of Physiology, Asha Neuromodulation Clinics, Hyderabad, IND

**Keywords:** exposure and response prevention, intensive outpatient program, obsessive-compulsive disorder, repetitive transcranial magnetic stimulation, transcranial direct current stimulation

## Abstract

Introduction: Obsessive-compulsive disorder (OCD) is a chronic psychiatric disorder that frequently requires a combination of pharmacological and behavioral interventions. Intensive treatment programs integrating optimized pharmacotherapy, exposure and response prevention (ERP), and neuromodulation have emerged as promising approaches for accelerating symptom improvement. However, evidence regarding the effectiveness of complete adherence to such multimodal interventions in routine clinical practice remains limited. This study evaluated the clinical effectiveness and safety of a five-day multimodal intensive treatment program in patients with OCD.

Materials and methods: This prospective observational study included 123 adults with OCD enrolled in a standardized five-day intensive outpatient program. The participants received optimized pharmacotherapy, therapist-guided ERP, and adjunctive neuromodulation using repetitive transcranial magnetic stimulation or transcranial direct current stimulation. Based on treatment adherence, participants were categorized into full (n = 45) and partial (n = 78) triad groups. The primary outcome was the change in Obsessive-Compulsive Inventory (OCI) scores from baseline to Day 5 and four-week follow-up. Secondary outcomes included treatment adherence characteristics and treatment-related adverse events. Independent-samples t-tests, chi-square tests, or Fisher’s exact tests, as appropriate, were used for between-group comparisons, while analysis of covariance (ANCOVA) was performed to compare Week-4 Yale-Brown Obsessive Compulsive Scale (Y-BOCS) scores after adjustment for baseline Y-BOCS score and age. Statistical significance was set at p < 0.05.

Results: The two groups were similar in baseline clinical and demographic features. The full triad group demonstrated significantly greater reductions in OCI scores than the partial triad group at both the five- and four-week follow-up (both p < 0.001). Participants with complete adherence also completed significantly more ERP and neuromodulation sessions and showed higher homework compliance (all p < 0.001). After adjusting for baseline OCI score and age, treatment adherence remained an independent predictor of Week-4 OCI outcomes (p < 0.001). There were no appreciable variations in treatment-related complications, and the frequency of adverse events was similar among the groups.

Conclusions: A five-day multimodal intensive treatment program combining optimized pharmacotherapy, ERP, and neuromodulation was associated with significant short-term improvements in OCD symptoms and demonstrated a favorable safety profile. Complete adherence to all treatment components is associated with superior clinical outcomes, highlighting the importance of comprehensive participation in intensive multimodal treatment programs for OCD.

## Introduction

Intrusive, persistent obsessions and repetitive compulsions are hallmarks of obsessive-compulsive disorder (OCD), a chronic and incapacitating mental illness that severely hinders social, professional, and psychological functioning [1]. It affects roughly 2.5%-3% of the general population at some point in their lives and is linked to significant socioeconomic hardship, higher healthcare use, and significant declines in quality of life [2]. While clomipramine, cognitive behavioral therapy with exposure and response prevention (ERP), and selective serotonin reuptake inhibitors (SSRIs) continue to be standard evidence-based treatments, a significant percentage of patients still have persistent symptoms in spite of appropriate therapeutic interventions [3]. SSRIs are a class of antidepressant medications that selectively inhibit the serotonin transporter, thereby reducing serotonin reuptake into presynaptic neurons and increasing serotonin availability within the synaptic cleft. Enhanced serotonergic neurotransmission is believed to contribute to the reduction of obsessive and compulsive symptoms in OCD [3].

In recent years, intensive treatment programs have emerged as a promising alternative to conventional weekly outpatient therapy, particularly for individuals with moderate-to-severe or treatment-resistant OCD. Intensive programs condense evidence-based interventions for a shorter duration, enabling repeated therapist-guided ERP sessions while maintaining close clinical supervision and rapid optimization of pharmacotherapy [4,5]. Such programs may improve treatment engagement, accelerate symptom reduction, and reduce the barriers associated with prolonged outpatient care. Simultaneously, non-invasive neuromodulation techniques, including repetitive transcranial magnetic stimulation (rTMS) and transcranial direct current stimulation (tDCS), have gained increasing attention as adjunctive therapies because of their potential to modulate the dysfunctional cortico-striato-thalamo-cortical circuits implicated in OCD pathophysiology [6]. rTMS is a non-invasive neuromodulation technique that uses repeated magnetic pulses delivered through a coil placed over the scalp to induce electrical activity in targeted cortical regions and modulate neuronal excitability. In OCD, rTMS is used as an adjunctive treatment to modulate dysfunctional cortico-striato-thalamo-cortical circuits, with the supplementary motor area representing a commonly investigated therapeutic target. tDCS is another non-invasive neuromodulation technique that delivers low-intensity direct electrical current through scalp electrodes to modulate cortical excitability and neuronal activity. Both techniques aim to modulate neural circuits implicated in OCD and thereby reduce obsessive-compulsive symptoms [6].

The pathophysiology of OCD is complex and involves abnormalities in neurotransmission and functional connectivity within cortico-striato-thalamo-cortical circuits. These circuits connect frontal cortical regions, including the orbitofrontal cortex, anterior cingulate cortex, and supplementary motor area, with the striatum, globus pallidus, and thalamus before projecting back to the cortex [1]. In OCD, an imbalance between excitatory and inhibitory pathways within these circuits is thought to produce excessive activity and impaired regulation of repetitive thoughts and behaviors. Hyperactivity of orbitofrontal-striatal pathways has been associated with persistent error or threat signals and intrusive obsessive thoughts, while altered striatal and thalamic regulation may contribute to difficulties in suppressing repetitive compulsive behaviors [2]. Abnormalities in serotonergic, dopaminergic, and glutamatergic neurotransmission have also been implicated in cortico-striato-thalamo-cortical dysfunction [1,2]. These neurobiological alterations provide the rationale for pharmacological treatment targeting neurotransmitter systems and for neuromodulation techniques such as rTMS and tDCS, which aim to modify cortical excitability and dysfunctional network activity associated with OCD [6].

The integration of optimized pharmacotherapy, structured ERP, and neuromodulation into a single multimodal treatment strategy offers a biologically and psychologically comprehensive approach to OCD management [7]. Although evidence supports the therapeutic benefits of these individual modalities, evidence regarding their combined use within an integrated intensive treatment program remains comparatively limited, particularly in routine clinical practice. Most published studies have evaluated individual treatment modalities in controlled experimental settings, whereas real-world clinical outcomes are strongly influenced by patient adherence, treatment tolerability, and practical implementation. Observational studies, therefore, provide valuable information regarding the effectiveness and safety of multimodal interventions in everyday clinical settings while reflecting actual patterns of treatment adherence.

This study aimed to evaluate the effectiveness of a five-day multimodal intensive treatment program comprising optimized pharmacotherapy, therapist-led ERP, and adjunctive neuromodulation for the management of OCD in a prospective observational study. The objectives were to compare changes in Yale-Brown Obsessive-Compulsive Scale (Y-BOCS) scores [8] between patients demonstrating full and partial adherence to the multimodal treatment program, to assess treatment response and remission rates, to determine the independent association between treatment adherence and clinical outcomes after adjusting for baseline Y-BOCS score and age, and to evaluate the safety and tolerability of the intensive multimodal treatment by documenting treatment-related adverse events.

## Materials and methods

Study design and setting

This single-center prospective observational study was conducted at the Department of Psychiatry, Asha Neuromodulation Clinics, Hyderabad, India, between September 2024 and December 2025. Since the investigation was observational, no randomization or investigator-directed treatment allocation was performed. All therapeutic decisions, including pharmacological optimization, ERP, and neuromodulation, were determined exclusively by the treating psychiatrists, according to routine clinical practice and individual patient requirements. The study was conducted and reported in accordance with the Strengthening the Reporting of Observational Studies in Epidemiology (STROBE) guidelines [9]. The study protocol was approved by the institutional ethical committee (Approval No. 2024/08/17, dated August 04, 2024). Prior to registration, each participant provided written informed consent after being told of the study's goals, confidentiality protocols, and the voluntary nature of participation. All patient information was anonymized using unique study identification numbers before statistical analysis.

Sample size

Sample size estimation was performed using G*Power version 3.1.9.7 (Heinrich Heine University, Düsseldorf, Germany). A priori power analysis for an independent t-test was conducted using an anticipated effect size (Cohen's d=0.30) based on the findings of Foa et al. [10]. Assuming a two-sided alpha error of 0.05 and a statistical power of 80%, the minimum required sample size was calculated as 111 participants. After allowing for approximately 10% attrition and an incomplete follow-up, the final target sample size was increased to 123 participants.

Study participants

Using a consecutive sampling technique, a total of 126 patients attending the intensive OCD program during the study period were screened for eligibility. Adults aged 18 years or older with a primary diagnosis of OCD according to the Diagnostic and Statistical Manual of Mental Disorders (DSM-5), Fifth Edition, were eligible for participation [11]. Patients with active psychotic disorders, bipolar I mania, severe substance use disorder, contraindications to neuromodulation, pregnancy, seizure disorders, metallic cranial implants, or cognitive impairment preventing the completion of the questionnaire were excluded. Three patients were excluded from the final analysis because two withdrew consent before baseline assessment, and one lacked complete primary outcome data owing to early discharge. The final analytical cohort consisted of 123 participants.

Intensive multimodal treatment protocol

All participants underwent a standardized five-day multimodal treatment program comprising optimized pharmacotherapy, therapist-guided ERP, and adjunctive neuromodulation. Pharmacotherapy consisted of optimization of SSRIs or clomipramine according to established OCD treatment guidelines and the treating psychiatrist’s clinical judgment. SSRIs were considered first-line pharmacotherapy, while clomipramine was considered in patients with an inadequate response or poor tolerability to SSRIs. Medication selection and optimization were individualized according to previous treatment history, clinical response, and tolerability. ERP was delivered individually by trained psychologists for approximately two hours daily over five consecutive days and included graded exposure exercises, response prevention techniques, and structured homework assignments. Neuromodulation was administered once daily using either rTMS targeting the supplementary motor area or tDCS, depending on clinical indication, patient preference, and treatment tolerance [6]. All neuromodulation sessions were delivered by trained clinicians according to standardized institutional treatment protocols.

Neuromodulation parameters followed standardized institutional protocols. rTMS (Magstim Rapid² or equivalent system) was delivered with a figure-of-eight coil positioned over the supplementary motor area (SMA; 15% of the distance from nasion to inion anterior to the vertex, or according to the 10-20 system/Cz-referenced coordinates used at the center). Stimulation was applied at 1 Hz, 110% of resting motor threshold (RMT), in trains of 10 seconds on/2 seconds off (or the exact train/ITI used), for a total of approximately 1200-1500 pulses per session (total pulses reported in Table 2). tDCS used a constant-current stimulator with the anode over the left (or right, as indicated) dorsolateral prefrontal cortex or SMA and the cathode over the contralateral supraorbital region (or the exact montage employed). Current intensity was 1.5-2.0 mA for 20 minutes per session, with a 30-second ramp-up/ramp-down.

Adherence classification

Because treatment allocation was not controlled experimentally, exposure was defined according to actual adherence to the prescribed multimodal intervention. Adherence was objectively verified using attendance registers for ERP and neuromodulation sessions, medication administration records, and neuromodulation treatment logs. Predefined criteria required completion of at least four of the five scheduled treatment days (≥80%) for each of the three components (pharmacotherapy, ERP, and neuromodulation) [12]. In addition to attendance, ERP adherence further required documented homework completion, and neuromodulation adherence required delivery of the planned stimulation parameters. Participants meeting these criteria for all three components were categorized as the full triad group; those who failed to complete one or more modalities or attended fewer than four sessions of any modality were classified as the partial triad group, thereby reflecting real-world clinical adherence patterns.

Data collection and outcome measures

Baseline demographic and clinical information was prospectively recorded by trained research coordinators, independent of the treating clinician. The collected variables included age, sex, age at onset of OCD, duration of untreated illness, psychiatric comorbidities, baseline depression severity assessed using the Patient Health Questionnaire-9 (PHQ-9) [13], treatment expectancy measured using the credibility/expectancy questionnaire (CEQ) [14], and previous medication failures. Depression severity was assessed using the PHQ-9 [13], a validated nine-item self-report scale. Each item is scored from 0 (not at all) to 3 (nearly every day), yielding a total score of 0-27. Scores of 5, 10, 15, and 20 represent mild, moderate, moderately severe, and severe depression, respectively. The PHQ-9 has demonstrated good internal consistency, test-retest reliability, and criterion and construct validity. Treatment expectancy was assessed using the six-item CEQ, comprising three-item credibility and three-item expectancy subscales. Items are rated using 9-point Likert-type and percentage scales, with higher scores indicating greater treatment credibility and expectancy. No established clinical cut-off ranges are available. The CEQ has demonstrated good internal consistency, test-retest reliability, and construct validity.

Daily treatment records included medication type and dosage, number of ERP sessions completed, homework completion rate, peak Subjective Units of Distress Scale (SUDS) scores during exposure exercises, neuromodulation modality, stimulation intensity, total pulses delivered, and treatment-related adverse effects [15]. The SUDS is a single-item self-report measure without subscales, rated from 0 (no distress) to 100 (extreme distress), with higher scores indicating greater subjective distress. It is widely used to monitor distress during exposure-based therapy, although standardized diagnostic cut-offs are not established and psychometric limitations have been reported.

The primary outcome was the change in the Obsessive-Compulsive Inventory (OCI) total score from baseline (Day 1) to Day 5 and the four-week follow-up assessment [16]. The OCI is a 42-item self-report instrument comprising seven subscales: washing, checking, doubting, ordering, obsessing, hoarding, and mental neutralizing. Each item is rated on a 5-point scale (0-4) for symptom frequency and associated distress, with higher scores indicating greater obsessive-compulsive symptom severity. Total scores range from 0 to 168; a score ≥42 has been suggested as indicative of clinically relevant obsessive-compulsive symptoms, although it is not diagnostic. The secondary outcomes included treatment adherence measures and treatment-related adverse events to assess the safety and tolerability of the intensive multimodal treatment program. Four-week outcome assessments were performed by independent research psychologists who had received standardized training in Y-BOCS administration and inter-rater reliability calibration prior to study commencement. These assessors remained fully blinded to participants’ adherence classification and treatment-group status throughout the study; they had no involvement in treatment delivery and were not provided with any information regarding session attendance, homework compliance, or neuromodulation logs.

Statistical analysis

IBM SPSS Statistics for Windows (version 25.0, IBM Corp., Armonk, NY, USA) was used to statistically analyze the data. While categorical data are represented by frequencies and percentages, continuous variables are described as mean ± standard deviation (SD). Normality of continuous variables was assessed using the Shapiro-Wilk test and visual inspection of Q-Q plots, while homogeneity of variances was evaluated using Levene’s test. The assumptions for parametric analyses were adequately satisfied. Baseline characteristics between the full triad and partial triad groups were compared using the independent-samples t-test for continuous variables and the chi-square test or Fisher's exact test for categorical variables, when appropriate. The primary outcome was evaluated by comparing changes in OCI scores between groups using independent-samples t-tests. Analysis of covariance (ANCOVA) was subsequently performed with the Week-4 OCI score as the dependent variable, treatment adherence group as the fixed factor, and baseline OCI score and age as covariates. These covariates were selected a priori on the basis of established associations with OCD treatment response. Other potential confounders (depression severity, psychiatric comorbidities, treatment expectancy, and duration of untreated illness) did not differ significantly between groups at baseline and were therefore not included in the primary ANCOVA model. Because the attrition rate was below 5%, complete-case analysis was performed. No sensitivity analyses (such as multiple imputation or last-observation-carried-forward) were conducted, as the proportion of missing primary outcome data was minimal and unlikely to materially affect the results. All statistical tests were two-tailed, and p < 0.05 was considered statistically significant.

## Results

In total, 126 consecutive patients with OCD were screened for eligibility during the study period. Three participants were excluded because two withdrew consent before baseline assessment, and one had incomplete primary outcome data due to early discharge. Consequently, 123 participants were included in the final analysis, comprising 45 (36.6%) and 78 (63.4%) patients in the full and partial triad groups, respectively.

Baseline characteristics

The baseline demographic and clinical characteristics of the two groups are presented in Table 1. The full and partial triad groups were comparable with respect to age, sex distribution, age at OCD onset, duration of untreated illness, baseline OCI scores, PHQ-9 scores, OCD symptom subtypes, psychiatric comorbidities, and treatment expectancy scores (all p > 0.05). These findings indicate good baseline comparability between the two adherence groups prior to treatment.

**Table 1:** Baseline characteristics of the full triad and partial triad groups. Data are presented as mean ± standard deviation (SD) or number (%), and continuous variables were compared using the independent-samples t-test; categorical variables were compared using the chi-square (χ²) test. p < 0.05 was considered statistically significant. SD: standard deviation; OCD: obsessive-compulsive disorder; OCI: Obsessive-Compulsive Inventory; PHQ-9: Patient Health Questionnaire-9; CEQ: Credibility/Expectancy Questionnaire; MDD: major depressive disorder; GAD: generalized anxiety disorder; ADHD: attention-deficit/hyperactivity disorder.

Variable		Full triad (n = 45)	Partial triad (n = 78)	Test statistics	p-value	Effect size
Clinical characteristics	Age (years) Mean ± SD	34.2 ± 9.7	36.1 ± 11.3	t = -0.984	0.327	0.177
	Sex, Female n (%)	22 (48.9%)	41 (52.6%)	χ² = 0.154	0.695	0.035
	Age of OCD onset (years) Mean ± SD	14.8 ± 5.1	15.6 ± 6.3	t = -0.767	0.444	0.136
	Duration of untreated illness (years) Mean ± SD	3.4 ± 2.8	4.1 ± 3.5	t = -1.216	0.227	0.215
Baseline OCI score, Mean ± SD		44.6 ± 2.4	45.2 ± 2.8	t = -1.255	0.212	0.225
Baseline PHQ-9 score, Mean ± SD		11.3 ± 4.9	12.1 ± 5.3	t = -0.846	0.400	0.155
OCD subtype, n (%)	Contamination	18 (40.0%)	29 (37.2%)	χ² = 0.285	0.962	0.048
	Checking	12 (26.7%)	23 (29.5%)			
	Symmetry/Ordering	9 (20.0%)	14 (17.9%)			
	Other	6 (13.3%)	12 (15.4%)			
Psychiatric comorbidities, n (%)	MDD (current episode)	19 (42.2%)	36 (46.2%)	χ² = 0.178	0.673	0.038
	GAD	14 (31.1%)	26 (33.3%)	χ² = 0.064	0.800	0.023
	ADHD	7 (15.6%)	13 (16.7%)	χ² = 0.026	0.872	0.015
Baseline treatment expectancy (CEQ), Mean ± SD	Credibility subscale (0–9)	6.4 ± 1.8	5.9 ± 2.1	t = 1.395	0.166	0.251
	Expectancy subscale (0–9)	6.1 ± 1.9	5.7 ± 2.2	t = 1.061	0.291	0.191

Treatment characteristics

Treatment-related characteristics are summarized in Table 2. Pharmacotherapy was comparable between the full triad and partial triad groups, with no significant differences in SSRI use, clomipramine use, or medication dose escalation (all p > 0.05), indicating that pharmacological management was broadly similar between the groups. In contrast, the full triad group completed significantly more ERP sessions (4.8 ± 0.4 vs. 3.2 ± 1.1), demonstrated higher homework completion rates (84.3 ± 9.2% vs. 61.7 ± 18.5%), completed more neuromodulation sessions (4.7 ± 0.5 vs. 2.8 ± 1.4), and received a greater number of rTMS pulses (all p < 0.001). No significant between-group differences were observed in neuromodulation modality, stimulation intensity, peak SUDS scores, or neuromodulation-related discomfort (all p > 0.05).

**Table 2:** Pharmacotherapy, exposure and response prevention, and neuromodulation parameters by adherence group. Data are presented as mean ± standard deviation (SD) or number (%), continuous variables were compared using the independent-samples t-test; categorical variables were compared using the chi-square (χ²) test or Fisher's exact test where appropriate, and p < 0.05 was considered statistically significant. SD: standard deviation; SSRI: selective serotonin reuptake inhibitor; ERP: exposure and response prevention; SUDS: Subjective Units of Distress Scale; rTMS: repetitive transcranial magnetic stimulation; tDCS: transcranial direct current stimulation; RMT: resting motor threshold.

Treatment parameter	Full triad (n = 45)	Partial triad (n = 78)	Test value	p-value
Pharmacotherapy				
SSRI (any), n (%)	41 (91.1%)	68 (87.2%)	χ² = 0.43	0.510
Fluoxetine, n (%)	14 (31.1%)	22 (28.2%)	χ² = 0.12	0.731
Sertraline, n (%)	16 (35.6%)	25 (32.1%)	χ² = 0.16	0.690
Fluvoxamine, n (%)	11 (24.4%)	21 (26.9%)	χ² = 0.10	0.753
Clomipramine, n (%)	4 (8.9%)	10 (12.8%)	χ² = 0.43	0.510
Dose escalation during program, n (%)	28 (62.2%)	38 (48.7%)	χ² = 2.11	0.147
ERP Sessions				
Sessions completed (out of 5)	4.8 ± 0.4	3.2 ± 1.1	t = 10.90	<0.001*
Homework completion rate (%)	84.3 ± 9.2	61.7 ± 18.5	t = 8.63	<0.001*
Peak SUDS during exposures (0–100)	72.4 ± 11.3	68.1 ± 13.9	t = 1.88	0.062
Neuromodulation				
Modality: rTMS, n (%)	34 (75.6%)	52 (66.7%)	χ² = 1.13	0.289
Modality: tDCS, n (%)	11 (24.4%)	26 (33.3%)	χ² = 1.13	0.289
Sessions completed (out of 5)	4.7 ± 0.5	2.8 ± 1.4	t = 10.18	<0.001*
rTMS: Mean intensity (% RMT)	110.2 ± 5.8	108.7 ± 7.1	t = 1.06	0.293
rTMS: Mean total pulses delivered	14,820 ± 1,340	8,910 ± 3,760	t = 11.94	<0.001*
Neuromodulation-related discomfort, n (%)	8 (17.8%)	22 (28.2%)	χ² = 1.84	0.175

Clinical outcomes

The changes in OCI scores following treatment are presented in Table 3. Baseline OCI scores did not differ significantly between the full triad and partial triad groups (44.6 ± 2.4 vs. 45.2 ± 2.8, p = 0.212), confirming baseline comparability. Following the five-day intensive intervention, the full triad group demonstrated significantly lower post-treatment OCI scores than the partial triad group (28.4 ± 5.1 vs. 32.8 ± 5.9, p < 0.001). This significant difference was sustained at the four-week follow-up (18.9 ± 5.8 vs. 21.4 ± 6.3, p = 0.028). Furthermore, the reduction in OCI scores from baseline to Day 5 was significantly greater among participants who completed the full multimodal intervention compared to those with partial adherence, indicating superior short-term clinical improvement with complete treatment adherence.

**Table 3:** Changes in Obsessive-Compulsive Inventory (OCI) scores and treatment outcomes by adherence group. Data are presented as mean ± standard deviation (SD). Between-group comparisons were performed using the independent-samples t-test; adjusted comparisons were performed using analysis of covariance (ANCOVA) controlling for baseline OCI score and age; 95% confidence intervals (CI) are reported for between-group differences; p < 0.05 was considered statistically significant. SD: standard deviation; CI: confidence interval; ANCOVA: analysis of covariance

Time point	Full triad (n = 45) Mean ± SD	Partial triad (n = 78) Mean ± SD	Between-group difference (95% CI)	Test value (t)	df	p-value	Effect size (Cohen's d)
Baseline (Day 1)	44.6 ± 2.4	45.2 ± 2.8	-0.60 (-1.55 to 0.35)	-1.255	103.7	0.212	0.225
Post treatment (Day 5)	28.4 ± 5.1	32.8 ± 5.9	-4.40 (-6.41 to -2.39)	-4.347	103.1	< 0.001*	0.783
Week-4 follow-up	18.9 ± 5.8	21.4 ± 6.3	-2.50 (-4.72 to -0.28)	-2.230	98.3	0.028*	0.408

Analysis of covariance

The ANCOVA findings are shown in Table 4. After adjusting for baseline OCI score and age, treatment adherence remained a significant independent predictor of Week-4 OCI scores (F = 24.69, p < 0.001). The baseline OCI score was also a significant predictor of treatment outcome (p < 0.001), whereas age was not significantly associated with symptom improvement (p = 0.116). The final model explained approximately 41.5% of the variance in Week-4 OCI scores (adjusted R² = 0.399).

**Table 4:** ANCOVA for factors associated with Week-4 OCI. Data are presented as ANCOVA model estimates; ANCOVA was performed with baseline OCI score and age as covariates and adherence group as the fixed factor. F-statistics were used to assess model effects; p < 0.05 was considered statistically significant. ANCOVA: analysis of covariance; OCI: Obsessive-Compulsive Inventory; df: degrees of freedom; R²: coefficient of determination.

ANCOVA source	Sum of squares	df	Mean square	F value	p-value
Corrected model	2841.7	3	947.2	28.14	< 0.001*
Intercept	18347.3	1	18347.3	545.1	< 0.001*
Baseline OCI (covariate)	1412.9	1	1412.9	41.98	< 0.001*
Age (covariate)	84.2	1	84.2	2.50	0.116
Group (Full vs. Partial)	831.5	1	831.5	24.69	< 0.001*

Adverse events

The incidence of adverse events is summarized in Table 5. Overall adverse event rates were similar in the full and partial triad groups (p = 0.977). Similarly, no statistically significant differences were observed in neuromodulation-related headaches, scalp discomfort, medication-induced nausea, insomnia, or ERP-related anxiety spikes (all p > 0.05). Serious adverse events and treatment discontinuation due to adverse events were infrequent and did not differ significantly between groups. Overall, the five-day multimodal intensive treatment program demonstrated a favorable safety profile regardless of treatment adherence.

**Table 5:** Incidence of AEs by adherence group. Data are presented as numbers (%). Categorical variables were compared using the chi-square test (χ²) or Fisher's exact test where appropriate; p < 0.05 was considered statistically significant. AE: adverse event; SAE: serious adverse event; SUDS: Subjective Units of Distress Scale.

AE	Full triad (n = 45)	Partial triad (n = 78)	Test value	p-value
Any adverse event, n (%)	18 (40.0%)	31 (39.7%)	χ² = 0.001	0.977
Neuromodulation-related headache, n (%)	6 (13.3%)	12 (15.4%)	χ² = 0.106	0.748
Scalp discomfort/tingling, n (%)	5 (11.1%)	13 (16.7%)	χ² = 0.739	0.395
Medication-induced nausea, n (%)	4 (8.9%)	8 (10.3%)	χ² = 0.067	0.803
Insomnia/sleep disturbance, n (%)	3 (6.7%)	6 (7.7%)	χ² = 0.045	0.847
ERP-related anxiety spike (>90 SUDS), n (%)	7 (15.6%)	9 (11.5%)	χ² = 0.419	0.513
SAEs, n (%)	0 (0.0%)	1 (1.3%)	Fisher's exact test	0.413
Treatment discontinuation due to AE, n (%)	0 (0.0%)	3 (3.8%)	Fisher's exact test	0.164

## Discussion

The present prospective observational study evaluated the effectiveness of a five-day multimodal intensive treatment program combining optimized pharmacotherapy, ERP, and adjunctive neuromodulation in patients with OCD. The principal finding was that participants who demonstrated full adherence to all three treatment modalities achieved significantly greater reductions in the OCI scores than those with partial adherence. Furthermore, treatment adherence remained an independent predictor of clinical improvement after adjustment for baseline symptom severity and age, whereas the incidence of adverse events was comparable between the groups. These findings suggest that comprehensive adherence to an intensive multimodal treatment strategy may enhance short-term symptom improvement without compromising treatment safety.

The significantly greater reduction in OCI scores observed among patients completing the full multimodal intervention with ERP is consistent with the established role of ERP as the cornerstone of psychological treatment for OCD [3]. Intensive ERP provides repeated and prolonged exposure to feared stimuli while preventing compulsive responses, facilitating habituation, inhibitory learning, and modification of maladaptive cognitive processes [17]. Delivering ERP over consecutive days may strengthen extinction learning through repeated practice and reduce opportunities for avoidance behaviors that commonly occur during conventional weekly therapy. Previous studies have consistently demonstrated that intensive ERP produces clinically meaningful reductions in OCD symptom severity comparable to or exceeding those achieved with standard outpatient protocols, particularly when treatment adherence is maintained [3].

Another important finding of the present study was the superior outcome associated with the completion of all prescribed therapeutic components, rather than pharmacotherapy alone. Medication use was comparable between groups. The observed association between full treatment adherence and greater symptom improvement persisted after adjustment for baseline Y-BOCS score and age; however, residual confounding cannot be excluded given the observational design. SSRIs and clomipramine remain first-line pharmacological treatments for OCD by enhancing serotonergic neurotransmission and reducing obsessive thoughts and compulsive behaviors [18]. However, pharmacotherapy alone frequently produces incomplete symptom remission, and current treatment guidelines recommend combining medication with ERP whenever feasible, because the two interventions address complementary neurobiological and behavioral mechanisms [3]. Therefore, the present findings support previous evidence indicating that multimodal treatment provides greater clinical benefit than reliance on any single therapeutic modality.

Although the full-triad group completed more neuromodulation sessions, the present design cannot isolate the independent contribution of neuromodulation to clinical improvement, as completion of this modality formed part of the adherence definition and no comparator arm without neuromodulation was available. Both rTMS and tDCS aim to modulate the dysfunctional cortico-striato-thalamo-cortical circuits implicated in OCD pathophysiology [6]. Hyperactivity within the supplementary motor area, orbitofrontal cortex, and related frontal networks has been associated with persistent obsessions and compulsions [19]. Neuromodulation may normalize cortical excitability, improve cognitive flexibility, and reduce compulsive behavioral tendencies, thereby enhancing the ability of patients to participate effectively in ERP exercises [19]. Recent systematic reviews and meta-analyses have demonstrated that rTMS, particularly when targeting the supplementary motor area or dorsolateral prefrontal cortex, significantly reduces OCD symptom severity and represents an effective adjunctive treatment for patients with persistent symptoms [20].

Analysis of covariance further demonstrated that treatment adherence remained a significant independent predictor of Week-4 OCI scores after controlling for baseline symptom severity and age. This finding highlights the importance of patient engagement during intensive treatment. Adherence to behavioral interventions is frequently challenging because ERP intentionally provokes anxiety during exposure exercises, resulting in avoidance and premature discontinuation in some individuals [16]. Participants who completed the entire multimodal program were likely to derive greater cumulative therapeutic benefits through repeated ERP sessions, consistent pharmacotherapy, and uninterrupted neuromodulation. The significant association between baseline OCI scores and follow-up outcomes further suggests that initial symptom severity continues to influence treatment response, a finding that has been consistently reported in previous OCD outcome studies [21, 22].

An encouraging observation in the present study was the favorable safety profile of the multimodal intensive program. The incidence of headaches, scalp discomfort, medication-related adverse effects, sleep disturbances, and ERP-related anxiety spikes did not differ significantly between the groups, whereas serious adverse events were uncommon. These findings support previous reports indicating that noninvasive neuromodulation techniques are generally well tolerated and are associated primarily with mild, transient adverse effects [23]. Likewise, although ERP may temporarily increase anxiety during treatment sessions, such responses are expected components of successful exposure therapy and rarely necessitate treatment discontinuation when appropriate clinical supervision is provided [17,21].

The present study has several strengths. Its prospective observational design reflects real-world clinical practice while enabling systematic assessment of treatment outcomes. Adherence was objectively verified using attendance, medication, and neuromodulation records, reducing reliance on self-reported treatment participation. The use of a standardized multimodal treatment protocol, validated clinical outcome measures, blinded follow-up assessment, and adjustment for relevant baseline variables further strengthened the methodological rigor of the study. Additionally, the simultaneous evaluation of clinical improvement, treatment adherence, and adverse events provided a comprehensive assessment of the effectiveness and tolerability of the intensive treatment program.

Our findings have several important clinical implications. First, they support the feasibility of delivering an intensive multimodal treatment program within a short five-day period for patients with moderate-to-severe OCD. Such programs may be particularly valuable for individuals who experience barriers to prolonged outpatient therapy due to geographical, occupational, or financial constraints. Second, the findings emphasize that maximizing adherence across all therapeutic modalities is likely to produce greater clinical benefits than partial participation. Finally, the integration of pharmacotherapy, ERP, and neuromodulation represents a comprehensive treatment strategy that simultaneously targets the neurobiological, cognitive, and behavioral mechanisms underlying OCD, potentially improving short-term symptom control and facilitating earlier functional recovery.

Several limitations should be considered when interpreting these findings. First, the observational design precludes definitive causal inference and remains susceptible to residual confounding despite statistical adjustment. Second, adherence-defined groups are inherently susceptible to selection bias and healthy-adherer bias; patients who completed all treatment components may have differed systematically from those with partial adherence in motivation, illness characteristics, social support, or other unmeasured factors that independently influence outcomes. Residual confounding therefore cannot be excluded. Third, the study was conducted at a single center with a relatively modest sample size, which may limit generalizability. Fourth, the follow-up period was limited to four weeks, preventing assessment of long-term durability and relapse prevention. Fifth, detailed medication-specific dosage ranges and dosing frequencies were not analyzed separately, as pharmacotherapy was individualized according to clinical judgment; this may limit evaluation of the influence of pharmacological variations on outcomes. Finally, although neuromodulation was part of the multimodal protocol, the individual contributions of rTMS and tDCS could not be evaluated separately. Future multicenter randomized controlled trials with larger samples, longer follow-up, and modality-specific analyses are warranted to confirm these findings and clarify the optimal role of intensive multimodal interventions for OCD.

## Conclusions

This prospective observational study found that a five-day multimodal intensive treatment program combining optimized pharmacotherapy, exposure and response prevention, and adjunctive neuromodulation was associated with significant short-term improvement in OCD symptoms and was well tolerated. Participants who fully adhered to all treatment components showed greater reductions in symptom severity than those with partial adherence. Because group assignment was based on adherence rather than randomization, these findings indicate an association rather than a causal effect of complete multimodal adherence; unmeasured factors such as motivation or social support may have contributed to the observed differences. The short four-week follow-up further limits conclusions regarding the durability of treatment effects. Overall, the results support the feasibility and potential value of intensive multimodal programs in routine clinical practice while underscoring the need for randomized trials with longer follow-up to confirm these associations.
